# Insights into *Acinetobacter baumannii* AMA205’s Unprecedented Antibiotic Resistance

**DOI:** 10.3390/ijms252111424

**Published:** 2024-10-24

**Authors:** German Matias Traglia, Fernando Pasteran, Samyar Moheb, Usman Akhtar, Sebastian Gonzalez, Carolina Maldonado, Nicholas Furtado, Ahmed Mohamed, Jenny Escalante, Marisel R. Tuttobene, Araceli Quillen, Claudia Fontan, Ezequiel Albornoz, Alejandra Corso, Robert A. Bonomo, Gauri G. Rao, Marcelo E. Tolmasky, Maria Soledad Ramirez

**Affiliations:** 1 Unidad de Genómica y Bioinformática, Departamento de Ciencias Biológicas, CENUR Litoral Norte, Universidad de la República, Salto 50000, Uruguay; gtraglia@litoralnorte.udelar.edu.uy; 2 National Regional Reference Laboratory for Antimicrobial Resistance (NRL), Servicio Antimicrobianos, Instituto Nacional de Enfermedades Infecciosas, ANLIS Dr. Carlos G. Malbrán, Buenos Aires C1282AFF, Argentina; fpasteran@gmail.com (F.P.); ealbornoz@anlis.gob.ar (E.A.); corsoalejandra@gmail.com (A.C.); 3 Center for Applied Biotechnology Studies, Department of Biological Science, College of Natural Sciences and Mathematics, California State University Fullerton, Fullerton, CA 92831, USA; mohebsamyar@csu.fullerton.edu (S.M.); uakhtar19@csu.fullerton.edu (U.A.); sebgonzalez@fullerton.edu (S.G.); carolina.dominguez@csu.fullerton.edu (C.M.); nifurtado@fullerton.edu (N.F.); ahmedmohamed@csu.fullerton.edu (A.M.); jp723670@gmail.com (J.E.); mtolmasky@fullerton.edu (M.E.T.); 4 Instituto de Biología Molecular y Celular de Rosario (IBR, CONICET-UNR), Rosario 2000, Argentina; tuttobene@ibr-conicet.gov.ar; 5 Hospital 4 de Junio, Dr. Ramon Carrillo, Roque Saenz Peña, Santiago del Estero H3700, Argentina; araceliguillen12@hotmail.com (A.Q.); clafontan@hotmail.com (C.F.); 6 Research Service and GRECC, Louis Stokes Cleveland Department of Veterans Affairs Medical Center, Cleveland, OH 44106, USA; robert.bonomo@va.gov; 7 Departments of Medicine, Pharmacology, Molecular Biology and Microbiology, Biochemistry, Proteomics and Bioinformatics, Case Western Reserve University School of Medicine, Cleveland, OH 44106, USA; 8 CWRU-Cleveland VAMC Center for Antimicrobial Resistance and Epidemiology (Case VA CARES), Cleveland, OH 44106, USA; 9 USC Alfred E. Mann School of Pharmacy and Pharmaceutical Sciences, University of Southern California, Los Angeles, CA 90089, USA; gaurirao@usc.edu

**Keywords:** *Acinetobacter*, CMY-6, carbapenemases, DNA acquisition, antibiotic resistance

## Abstract

The rise of antibiotic-resistant bacteria in clinical settings has become a significant global concern. Among these bacteria, *Acinetobacter baumannii* stands out due to its remarkable ability to acquire resistance genes and persist in hospital environments, leading to some of the most challenging infections. Horizontal gene transfer (HGT) plays a crucial role in the evolution of this pathogen. The *A. baumannii* AMA205 strain, belonging to sequence type ST79, was isolated from a COVID-19 patient in Argentina in 2021. This strain’s antimicrobial resistance profile is notable as it harbors multiple resistance genes, some of which had not been previously described in this species. The AmpC family β-lactamase *bla*_CMY-6_, commonly found in Enterobacterales, had never been detected in *A. baumannii* before. Furthermore, this is the first ST79 strain known to carry the carbapenemase *bla*_NDM-1_ gene. Other acquired resistance genes include the carbapenemase *bla*_OXA-23_, further complicating treatment. Susceptibility testing revealed high resistance to most antibiotic families, including cefiderocol, with significant contributions from *bla*_CMY-6_ and bla_NDM-1_ genes to the cephalosporin and carbapenem resistance profiles. The *A. baumannii* AMA205 genome also contains genetic traits coding for 111 potential virulence factors, such as the iron-uptake system and biofilm-associated proteins. This study underscores *A. baumannii*’s ability to acquire multiple resistance genes and highlights the need for alternative therapies and effective antimicrobial stewardship to control the spread of these highly resistant strains.

## 1. Introduction

During the last few years, the prevalence of antibiotic-resistant bacteria in clinical settings has dramatically risen worldwide, alarming scientists and government agencies. According to the Centers for Disease Control and Prevention’s (CDC) 2019 Antibiotic Resistance Threats Report, antibiotic-resistant bacteria and fungi cause more than 2.8 million infections and 35,000 deaths annually, with annual healthcare costs exceeding USD 281 million in the United States [1]. A pathogen of particular concern is *Acinetobacter baumannii*, a “difficult-to-treat” bacterium responsible for infections with mortality rates as high as 60% (through community-acquired pneumonia) and 43.4% (through bloodstream infections) [2]. *A. baumannii* remains a top priority in the latest WHO list and has been categorized as an “Urgent” threat by the CDC.

Intrinsic features such as *A. baumannii*’s ability to persist in clinical settings even under desiccation and nutrient starvation, as well as its ability to acquire foreign DNA, have contributed to its success as a major nosocomial pathogen [3,4,5,6]. The indiscriminate use of broad-spectrum antibiotics in hospital settings favors the selection of organisms harboring active mechanisms of horizontal gene transfer and extreme genome plasticity, such as *A. baumannii*. Comparative genomic studies have revealed high variability in *Acinetobacter* genome organization and the presence of foreign DNA sequences, suggesting that acquiring exogenous genetic traits has significantly contributed to the evolution and adaptation of the genus to unfavorable environmental conditions. Among numerous mechanisms that drive *A. baumannii* evolution, acquiring foreign DNA by natural transformation plays a key role but is often underestimated [4,7]. Recent research has shown that *A. baumannii* can acquire DNA and consequently, antibiotic resistance, including resistance to carbapenems from other *A. baumannii* clinical strains, *Klebsiella pneumoniae, Providencia rettgeri*, and methicillin-resistant *Staphylococcus aureus* [8]. The acquired DNA often includes mobile genetic elements, antimicrobial resistance genes, and operons involved in metabolism. In silico gene analysis indicates that when *A. baumannii* and *K. pneumoniae* share the same environment, they continuously exchange genetic material [8]. Furthermore, natural transformation is primarily responsible for recombination events between *A. baumannii* clinical isolates, leading to carbapenem resistance [7]. Recent research highlights the ongoing genomic plasticity and acquisition of new genetic material in *A. baumannii*. A comparative study by Traglia et al. (2023) [9] examined the genomes of two *A. baumannii* isolates, one collected prior to and one following the COVID-19 pandemic. Their analysis, which involved core-genome phylogeny and comparative genomic studies, revealed notable differences in the antimicrobial resistance profile of the post-pandemic strain. This strain had acquired eight additional antimicrobial resistance genes, including the *bla*_NDM-1_ gene, which significantly enhances its resistance to carbapenem antibiotics. These findings emphasize how *A. baumannii* can evolve and adapt in response to changing environmental pressures, such as those posed by the COVID-19 pandemic [9] (cita).

Although *A. baumannii* is known for its ability to acquire DNA fragments, certain genes remain host-specific and are rarely, if ever, found in this bacterium. For example, *A. baumannii* isolates seldom carry the carbapenemases KPC, SPM-1, and VIM-2, which are typically found in Enterobacterales and *Pseudomonas* spp. [10]. Other genes with strict host specificity include the CMY-like plasmid-encoded β-lactamase genes, which are exclusively found in Enterobacterales. These genes, with 190 reported variants (http://bldb.eu/alignment.php?align=C:CMY, accessed on 22 October 2024), are primarily located on IncC and IncI1 plasmids. Among this large group of CMY-like β-lactamase coding genes, CMY-1-like genes are the most prevalent plasmid-borne *ampC* genes in Enterobacterales [11,12,13]. IncI1 plasmids, often found in isolates from animals and hospitalized patients, have recently gained attention due to their ability to transfer between different bacteria. To date, *bla*_CMY-like_ genes have not been detected in *A. baumannii*.

In this study, we describe *A. baumannii* AMA205, a highly resistant clinical strain that harbors *bla*_CMY-6_ and multiple antibiotic resistance mechanisms, including *bla*_OXA-23_ and *bla*_NDM-1_.

## 2. Results and Discussion

### 2.1. Genomic and Phylogenomic Comparative Analysis of A. baumannii AMA205 Reveals a Distinct Location of ST79

**Figure 1:**
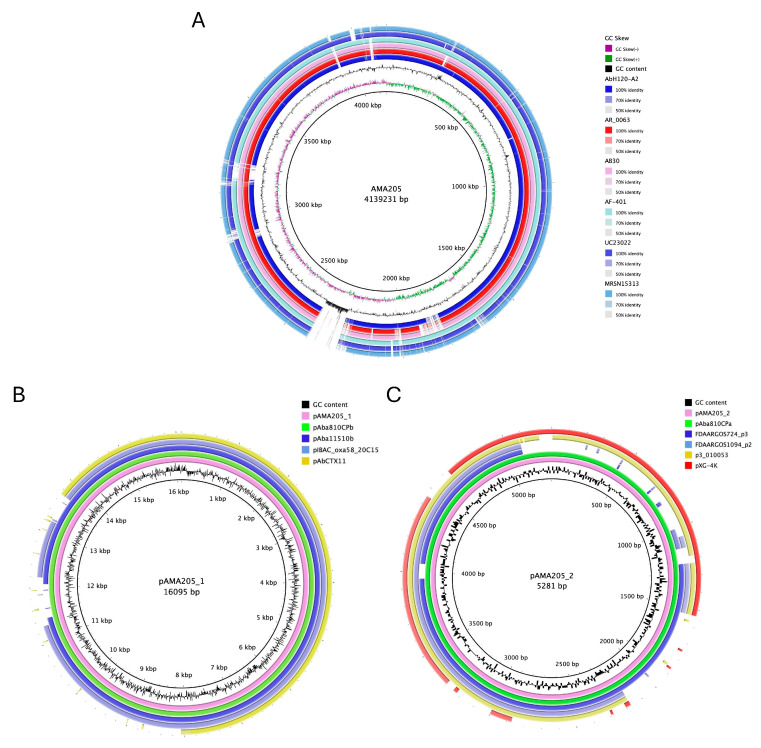
Comparative genomics circular visualization of AMA205. AMA205 used as reference in BRIGS. (**A**) AMA205 chromosome comparison against ST79 complete genome. The inner circle shows the GC content and skew of the reference sequence. Blast comparisons with strains are shown ordered according to the observed phylogenetic distance, from inside (close) to the outside rings: AbH12O, AR_0063, AB30, AF-401, UC23022, and MRSN15313. (**B**) pAMA205_1 comparison against closest related plasmid available in GenBank. The inner circle shows the GC content of the reference sequence. Blast comparisons with strains are shown ordered according to the nucleotide identity and coverage, from inside (close) to the outside rings: pAba810CPb, pAba11510b, pIBAC_oxa58_20C15, and pAbCTX11. (**C**) pAMA205_2 comparison against closest related plasmid available in GenBank. The inner circle shows the GC content of the reference sequence. Blast comparisons with strains are shown ordered according to the nucleotide identity and coverage, from inside (close) to the outside rings: pAba810CPa, FDAARGOS724_p3, FDAARGOS1094_p2, p3_010053, and pXG_4K.

The ANI% score (e.g., 98.2 ± 1.80% with ATCC 17978) confirmed that the AMA205 isolate belongs to the *A. baumannii* species. The hybrid assembly of the AMA205 strain sequence produced a 4.139.231 bp- chromosome with a G + C content of 39.06% and 4007 predicted protein-coding sequences with an average gene length of 900 bp (Table 1 and Figure 1A). Additionally, the procedure identified two plasmids, pAMA205_1 and pAMA205_2, whose sizes were 16.095 bp and 5.281 bp, respectively (Table 1 and Figure 1B,C). Comparing AMA205 plasmids with the GenBank database, pAMA205_1 and pAMA205_2 were found in other *A. baumannii* isolates with high coverage (90–100%) (Figure 1B,C). Interestingly, pAMA205_1 contains the *znuB* gene encoding a TonB-dependent receptor, which may play an important role in the virulence of AMA205.

Additionally, a comparison of the AMA205 genome with all 246 *A. baumannii* ST79 genomes deposited in the GenBank database (Table S1) revealed 1555 conserved gene families and 139 unique genes. The most clinically relevant unique genes belonged to mobile genetic elements, such as insertion sequences (IS*Ecp1*, IS*Kpn8*, IS*103*, etc.), and coded for the β-lactamases NDM-1 and CMY-6 (Table S2).

**Figure 2:**
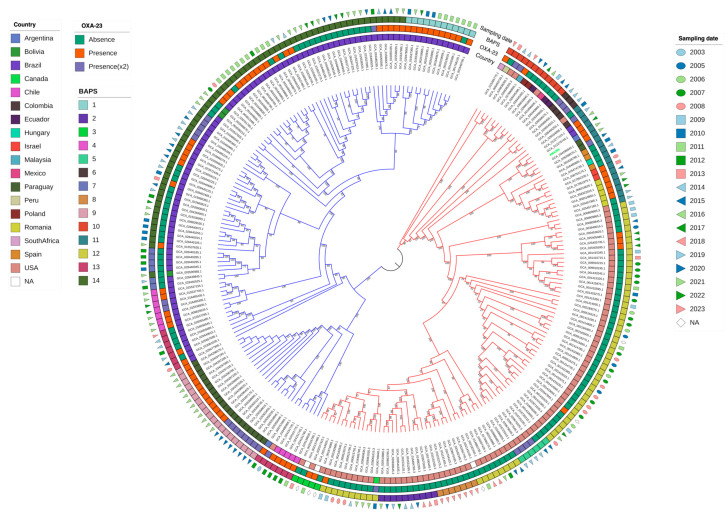
Core-genome phylogenetic analysis of AMA205 and 246 ST79 *A. baumannii* genomes. The figure displays the maximum likelihood phylogeny of 247 *A. baumannii* sequences. The bootstrap method was used as a supporting method (1000 iterations). The molecular substitution model was GTR. The tree representation was carried out by iTOL. Red and blue branches represent A and B phylogenetic clusters, respectively. The country of isolation, OXA-23 (absence/presence), BAPS1 cluster, and sampling date are provided for each strain.

A core-genome phylogenetic analysis including *A. baumannii* AMA205 and all ST79 *A. baumannii* genomes currently in the GenBank (n = 246), which includes strains isolated worldwide between 2003 and 2023 (Table S1), identified two major clusters: Cluster A and Cluster B. *A. baumannii* AMA205 was located in Cluster A, which included a diverse array of isolates from various regions worldwide, predominantly from the USA (90/123, 73.17%). Specifically, *A. baumannii* AMA205 was closely related to isolates from Paraguay and Brazil recovered during 2021–2022 (Figure 2). Cluster B primarily consisted of isolates from South America, except for one isolate from the USA (GCA_016486965.1). Most isolates in Cluster B were from Brazil (94/124, 75.81%) and Paraguay (20/124, 16.13%), while Cluster A was mainly composed of isolates from the USA (90/123, 73.17%).

Population structure was inferred using BAPS clusters defined at the first stratification level, capturing the dataset’s total genetic variation. This analysis divided the isolates into 14 BAPS clusters, with a significant correlation between the maximum likelihood phylogenetic tree and the BAPS clusters (Figure 2). Cluster A included eight BAPS clusters (2, 3, 5, 6, 8, 10, 11, 12), while Cluster B comprised six BAPS clusters (1, 4, 7, 9, 13, 14). *A. baumannii* AMA205 was placed within BAPS 11. These genetic clusters likely reflect common population differentiation processes, objectively defining groups of strains with similar genetic characteristics.

Further analysis revealed that the Brazilian isolates in Cluster B are divided into four populations (BAPS: 4, 7, 13, 14), while all Paraguayan isolates belong to a single population (BAPS: 9). Within the Paraguayan population, a correlation was observed between the presence of *bla*_OXA-23_, either in single or duplicated copies, and the year of isolation. Isolates from 2021 have a single *bla*_OXA-23_ copy, whereas those from 2020 have the gene duplicated. In contrast, most isolates in Cluster A lack the *bla*_OXA-23_ gene. This low prevalence is particularly evident in isolates from the USA, where the gene is present in 12 out of 90 isolates (13.3%). Another important observation is that none of the available genomes showed the presence of *bl_a_*_NDM-1_ in ST79. Therefore, AMA205 is the first *A. baumannii* strain with CMY-6 but also the first ST79 strain with the co-occurrence of *bla*_OXA-23_ and *bla*_NDM-1_.

**Figure 3:**
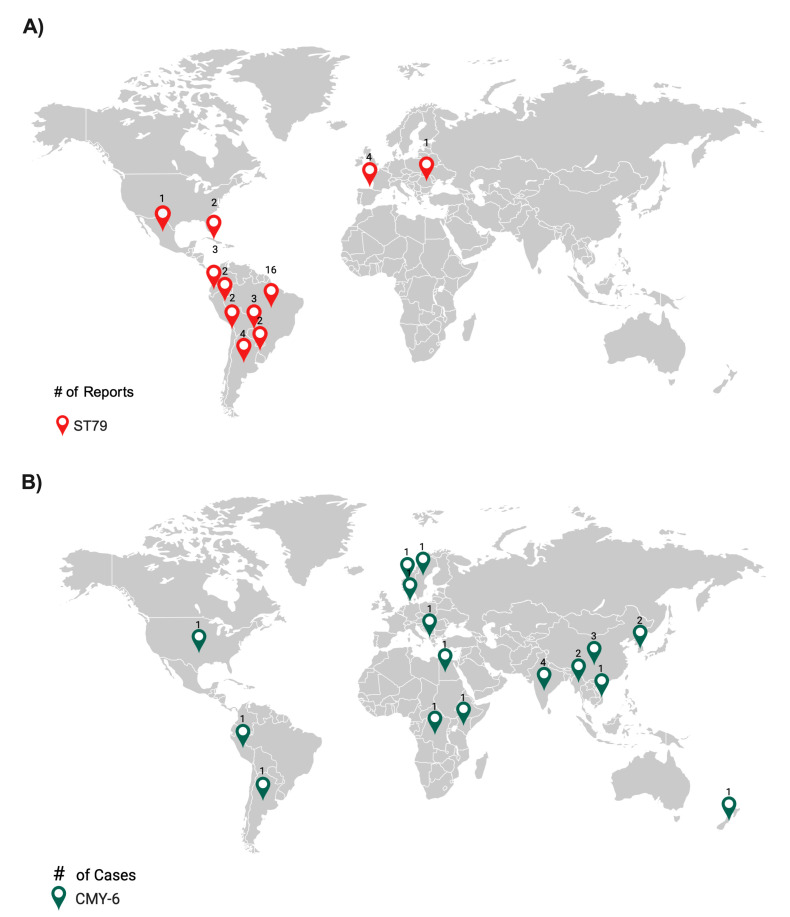
Comparative analysis of ST79 (**A**) and CMY-6 (**B**) distribution in clinical isolates from around the world. #: number of cases reported.

Most of the *A. baumannii* ST79 strains published to date (based on a PubMed search performed in June 2024) were from Latin America (n = 35, 87.5%), with additional cases in Spain (n = 4, 10.0%) and a single case in Romania (n = 1, 2.5%). The highest number of isolates were from Brazil (n = 16, 45.7%) (Figure 3A) (Table S3). Eighteen strains possessed *bla*_OXA-23_, of which eleven were from Brazil. These results suggest that the ST79 most likely originated in Latin America, where the earliest report occurred in 2014 and the most recent in 2022. Infections caused by the sequence type outside Latin America may be attributed to infected travelers.

### 2.2. AMA205 Exhibits Resistance to Almost All Tested Antibiotics, Including Cefiderocol

Susceptibility testing of AMA205 to various antibiotics, including cloxacillin (CX), ceftazidime (CAZ), ceftazidime–avibactam (CZA), imipenem (IMP), meropenem (MEM), ampicillin–sulbactam (AMS), amikacin (AK), gentamicin (CN), and tigecycline (TIG) was conducted. As anticipated, the clinical isolate exhibited high resistance to most antibiotic families tested (Table 2). The high MIC of cefiderocol (FDC), determined by two different methodologies, showed that AMA205 is also resistant to this antibiotic. Furthermore, the presence of intracolonies (IHC) was evident in this isolate when E-strips were used. The isolate demonstrated a high level of resistance to ceftazidime, with a MIC greater than 256 mg/L, which remained unchanged with the individual use of MBL inhibitors and cephalosporinases. However, when both inhibitors were used simultaneously, the MIC decreased to 16 mg/L. This result indicates that both *bla*_NDM-1_ and *bla*_CMY-6_ actively contribute to the cephalosporin resistance profile of this isolate.

The significant resistance of AMA205 is due to the presence of multiple resistance genes, including CMY-6, OXA-23, and NDM. This extensive array of resistance mechanisms not only complicates treatment options with currently approved drugs but also highlights critical knowledge gaps in our understanding of how these genes contribute to multidrug resistance in clinical settings. The global spread of such resistant strains poses a substantial threat to public health, underscoring the urgent need for alternative therapeutic approaches and robust antibiotic stewardship programs.

To address these challenges, efforts should be directed toward developing novel antimicrobials, such as combination therapies that target multiple resistance pathways simultaneously, and rapid diagnostic tools for detecting resistance genes. Additionally, enhanced surveillance programs are needed to monitor the emergence and dissemination of these resistance determinants, facilitating timely intervention and management strategies in both local and international healthcare settings. The findings emphasize that without a coordinated global response, the spread of strains like AMA205 may continue to escalate, further limiting treatment options and compromising patient outcomes.

### 2.3. Genomic Studies Reveal the Presence of CMY-6 and Other Antimicrobial Resistance Genes in the AMA205 Genome

The genomic analysis of *A. baumannii* AMA205 revealed antibiotic resistance genes within its core (intrinsic genes) and accessory (acquired genes) genomes. The acquisition of genetic determinants, a critical factor in *A. baumannii*’s evolution, occurs through mechanisms like transformation, conjugation, and transduction, involving mobile genetic elements [15,16]. Genes conferring resistance to chloramphenicol (*cmlB1*), florfenicol (*floR*), β-lactams (*bla*_OXA-23_, *bla*_OXA-65_, *bla*_ADC-25_, *bla*_CMY-6_, *bla_TEM-1B_*, and *bla*_NDM-*1*_), aminoglycosides (*aac(6′)-Ib3-like*, *rmtC*, *aac(6′)-Ian*, *strA*, and *strB*), and sulfonamides (*sul1* and *sul2*) were identified. The intrinsic genes *bla*_ADC-25_ and *bla*_OXA-65_ were present, but not associated with flanking insertion sequences (ISs), which is linked to their basal expression, resulting in weak levels of β-lactam hydrolysis [17,18]. All other antibiotic resistance genes were flanked by mobile genetic elements, suggesting acquisition through horizontal gene transfer. The globally distributed *bla*_OXA-23_ gene, found in both chromosomes and plasmids, was located within the transposon, Tn*2008*. This transposon, along with Tn*2006*, is one of the most common platforms harboring *bla*_OXA-23_. Although these transposons are typically associated with a *TnAbaR4-like* island [19,20,21], in *A. baumannii* AMA205, Tn*2008* was found outside the *TnAbaR-like* element. A β-lactamase gene *bla*_TEM-1B_ was identified within Tn*3*, differing from the usual association with the Tn*AbaR* element seen in other *A. baumannii* isolates where blaTEM-1B has been detected.

**Figure 4:**
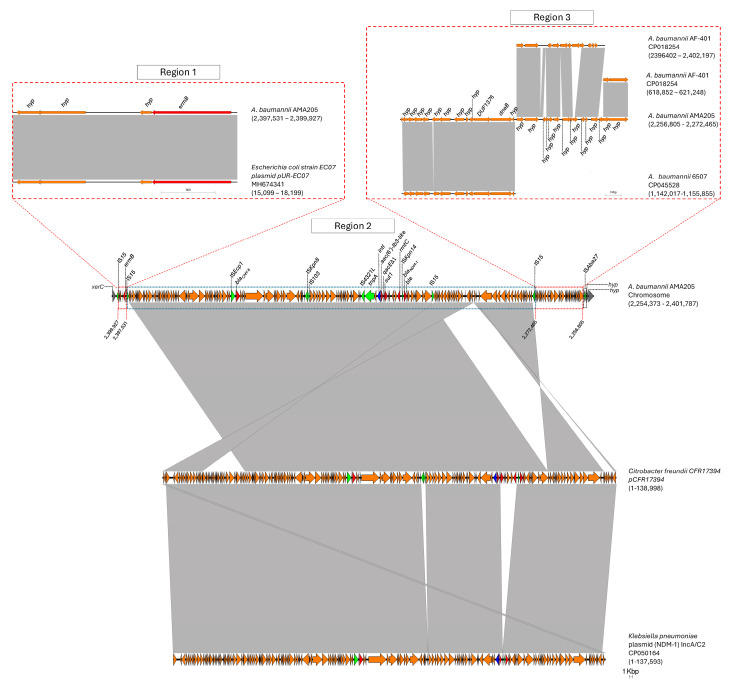
Comparison of genetic structure of GI-CMY genomic island. Gray bars, regions shared between isolates; red arrows, antibiotic resistances genes; green arrows, IS elements; blue arrows, class I integrase. The figure was created using EasyFig, version 2.2.2.

A large genomic island (GI-CMY) was identified in the AMA205 strain (Figure 4). This 147.4-kb sequence is flanked by the *xerC* gene, which encodes the XerC recombinase protein, and a gene for a hypothetical protein. The GI-CMY contains genes that confer resistance to aminoglycosides (*aac(6′)-Ib3-like, rmtC*, and *ermB*), β-lactams (*bla*_CMY-6_ and *bla*_NDM-1_), and sulfonamides (*sul1*) (Figure 4). Based on sequence homology, three regions were identified within GI-CMY. Region 1 harbors a 2396 bp fragment containing four genes (three encoding hypothetical proteins and the *emrB* gene) that show high homology to a fragment of the *Escherichia coli* plasmid pUR-EC07 (Coverage: 100%, Nucleotide Identity: 100%). Region 2, the largest at 125,066 bp, shows high homology with various Enterobacterales plasmids, such as those from *Citrobacter freundii* (Coverage: 99, Nucleotide Identity 100) and *Klebsiella pneumoniae* (Coverage: 93%, Nucleotide Identity: 99%). Notably, an important region of the chromosomal insertion of the *bla_CMY-6_* gene in AMA205 resembles the plasmid previously documented by Martino et al. [22]. This region contains a class 1 integron with *aac(6′)-Ib3-like* in the variable region and also includes the cephalosporin resistance gene *bla*_CMY-6_ and the carbapenemase *bla*_NDM-1_, located downstream of the insertion sequences IS*Ecp1* and IS*Kpn14*, respectively (Figure 4).

The AAC(6′)-Ib3-like variant identified shows an amino acid change at the first position (M1L), defining it as a new variant of aminoglycoside 6′N-acetyltransferase type Ib. The activity spectrum of this new variant will be investigated in future studies. Finally, region 4 (15,660 bp) contains fragments of *A. baumannii* sequences that show greater homology with genomes from other ST (6507) or more distantly related strains within the same sequence type (AF-401). These findings suggest that GI-CMY has undergone at least four to five recombination events, two of which likely involve the integration of DNA sequence segments commonly found in Enterobacterales (regions 1 and 2). It is hypothesized that two intra-species recombination events may have occurred in region 3. The recombination of these regions and the potential mobilization of GI-CMY may be facilitated by the various ISs flanking each region and the overall structure of the genomic island (IS*15* and IS*Aba27*).

As of June 2024, the *bla*_CMY-26_ gene was reported worldwide; more than half of the cases were from Asia (n = 12, 54.5%) and two from Latin America (9.1%) (Figure 3B). This gene is frequently found alongside *bla*_NDM-1_ in various species belonging to Enterobacterales, including *E. coli*, *K. pneumoniae*, and *Providencia vermicola*.

To investigate the frequency or rarity of the *bla*_CMY_ gene acquisition, a search was conducted of 120 CRAB clinical isolates, all of which tested negative. This finding suggests that the *bla*_CMY_ gene is rare in this species.

The AMA205 strain, which shows resistance to multiple antibiotics, is also resistant to cefiderocol. The resistance is linked to mutations in genes involved in iron uptake, such as *pirA, piuA*, and *cirA* [23,24]. A comparison of the *pirA* and *piuA* genes from AMA205 with those from the cefiderocol-susceptible reference strain ATCC17978 revealed 100% amino acid identity, indicating that mutations in these genes are not responsible for resistance to cefiderocol. However, the *cirA* from AMA205 showed 87% amino acid identity of 87% with 100% coverage compared to ATCC17978, including a significant deletion of six nucleotides in the gene sequence. These differences in the *cirA* gene, along with the presence of multiple β-lactamases, may partially explain the cefiderocol resistance phenotype.

### 2.4. AMA205 Genomic Analysis Revealed the Presence of a Variety of Virulence Factors

Recent research identified virulence factors in *A. baumannii* [25]. Using the VFDB database, 111 potential virulence factors coding genes were found in the *A. baumannii* AMA205 strain (Table S4).

Adherence to the host cell is the crucial initial step in bacterial colonization and infection. During this process, bacteria can form microcolonies that develop into a highly organized microbial community known as a biofilm. In *A. baumannii*, the initial stage of biofilm formation is driven by elements coded for by the *csuA/BABCDE* operon genes. A key factor in this process is the fimbriae chaperone, which is responsible for the assembly and production of pili that facilitate surface adhesion [26,27]. The regulation of this operon is controlled by a two-component system (BfmRS), comprising a kinase sensor (BfmS) and a response regulator (BfmR) [28,29]. The development of a mature biofilm structure involves a biofilm-associated protein (Bap), an ortholog of the protein found in Staphylococcus species, first identified in the *A. baumannii* AB307-0294 strain [30,31]. This study confirmed the presence of the *csuA/BABCDE* operon, the *bfmSR* regulatory system, and the bap gene in the *A. baumannii* AMA205 genome (Table S4).

The functions of TonB, ExbB, and ExbD are not limited to the acinetobactin iron uptake system. These three inner membrane proteins are involved in transporting various molecules, including heme, vitamin B12, and other iron-siderophore complexes [32,33,34,35]. Additionally, three distinct copies of *tonB* have been identified, *tonB1* and *tonB3*, which, along with *exbB* and *exbD*, form an operon, and *tonB2*, a monocistronic gene. All five genes were found in the *A. baumannii* AMA205 genome.

*A. baumannii* AMA205 also contains the bfn locus, which includes genes responsible for the biosynthesis of baumannoferrin, a siderophore first found in the *A. baumannii* AYE strain [26,35]. Baumannoferrin has a higher affinity for iron than acinetobactin. Its synthesis and internalization operate independently of the genes specific to the acinetobactin iron uptake system. The similarity of the Bfn proteins in *A. baumannii* AMA205 to those in other Acinetobacter species, along with the fact that the locus is not ubiquitous to *A. baumannii* [36,37], suggest that it was acquired through horizontal gene transfer (Table S4).

The capsular polysaccharide is a crucial virulence factor in Gram-negative bacteria, enabling resistance to the bactericidal activity of the complement system. *A. baumannii* AMA205 contains the capsular polysaccharide biosynthesis loci (KL, K locus) and LPS loci (OCL, OC locus). These loci are typically genomic “hotspots” of variability [38,39]. Comparative analysis of the KL structure in *A. baumannii* AMA205 showed a GC content of 33.35%, 99% nucleotide identity, and 100% coverage with the KL9 type (Figure sup KL and OC). The OCL locus, responsible for O antigen synthesis, had a GC content of 36.01% and was identified as OCL10-like (Table S4).

## 3. Materials and Methods

### 3.1. Bacterial Isolates

*A. baumannii* AMA205 was isolated in 2021 (Argentina) from a 30-year-old patient admitted to hospital with COVID-19, who developed sepsis and pneumonia after 11 days of hospitalization. The AMA205 strain was cultured in Luria Bertani (LB) medium and was initially identified using MALDI-TOF MS [29]. The identification was later confirmed by whole-genome sequencing (WGS) analysis. In addition, 120 CRAB strains from the National Regional Reference Laboratory for Antimicrobial Resistance (ANLIS–Dr. Carlos G. Malbrán) collection were used to search for the presence of *bla*_CMY_ by PCR amplification using primers CMY-F 5′-TGGCCAGAACTGACAGGCAAA-3′ and CMY-R 5′-TTTCTCCTGAACGTGGCTGGC-3′.

### 3.2. Whole Genomic Sequencing (WGS)

AMA205 DNA was extracted using the Wizard^®^ Genomic DNA Purification Kit (Madison, WI, USA) according to the manufacturer’s protocol. Whole genome sequencing (WGS) was conducted using the Illumina NovaSeq X Plus (San Diego, CA, USA) sequencer platform and Oxford Nanopore MinION Mk1B (San Francisco, CA, USA) (Seqcenter sequencing service). Sequencing quality was evaluated using FASTQC software (version 0.12.0)(https://www.bioinformatics.babraham.ac.uk/projects/fastqc/, accessed on 22 October 2024). De novo assembly was performed with Unicycler (version 0.5.1) (https://github.com/rrwick/Unicycler, accessed on 22 October 2024) and quality assessment was conducted using QUAST software (version 5.2). The genome annotation files can be found in the zenodo repository (https://zenodo.org/records/13741979, accessed on 22 October 2024). The Whole Genome Shotgun project has been deposited in GenBank with accession numbers CP169298 (AMA205 chromosome), CP169299, and CP169300 (plasmids pAMA205_1 and pAMA205_2, respectively).

### 3.3. Comparative Genomic Analysis

The AMA205 genome was annotated using PROKKA (version 1.14.5) [40]. The ortholog functional assignment was performed using EggNOG v2.0 (default parameter) [40]. To validate the species identification, the average nucleotide identity (ANI) was calculated using JSpeciesWS (version 4.2.1) [41] and reference genomes of Acinetobacter available in the NCBI genome database. To assess core genome phylogeny, we used 246 *A. baumannii* ST79 sequences from a total of 25,087 *A. baumannii* genomes available in the GenBank (Table S1). Core genome phylogeny analysis was performed using the maximum likelihood method, implemented with IQtree2 using default parameters [42].

Bayesian Analysis of Population Structure (BAPS) was performed using the “fastbaps” R package (version 1.0.8) [43] (https://github.com/gtonkinhill/fastbaps/, accessed on 22 October 2024). This software employs a phylogeny-independent, nested Bayesian clustering method to analyze population stratification, using core-genome sequences as input data.

tRNA and ncRNA predictions were conducted using tRNAscan-SE (version 1.3) and Infernal (version 1.1.5) software, respectively [44], and the Multilocus Sequence Typing (MLST) profile was determined using MLST scripts (URL: https://github.com/tseemann/mlst, accessed on 22 October 2024). AMA205 genomic DNA was extracted using the Wizard^®^ Promega kit (Promega, Madison, WI, USA). Antimicrobial resistance and virulence genes were identified using VFDB and Resfinder databases [45,46], respectively, using the BLASTp (version 2.2.25) software. 

### 3.4. Antibiotic Susceptibility Testing (AST)

AST profiles of AMA205 were performed following the Clinical and Laboratory Standards Institute (CLSI) guidelines as described in the 30th Edition informational supplement [14]. Disk diffusion was firstly performed with the following antibiotics: 10 µg ampicillin/sulbactam, 30 µg amikacin, 30 µg cefepime, 30 µg ceftazidime 5 µg ciprofloxacin, 10 µg imipenem, 10 µg gentamicin, 10 µg meropenem, 15 µg tigecycline, 30 µg minocycline, or 10 µg colistin. Broth microdilution for minimum inhibitory concentration (MIC) determination was conducted according to CLSI guidelines. For cefiderocol susceptibility, three different methods, commercial E-strips (Liofilchem S.r.l., Roseto degli Abruzzi, Italy), ComASP ((Liofilchem S.r.l.), and broth microdilution (reference method), were used. The methods were performed according to the manufacturer’s instructions and EUCAST standards (https://www.eucast.org/clinical_breakpoints, accessed on 22 October 2024).

Each experiment was repeated at least three times for each strain. Results were interpreted using CLSI guidelines, except for colistin and tigecycline, which were interpreted using the European Committee on Antimicrobial Susceptibility Testing (EUCAST) and Food and Drug Administration (FDA) recommendations, respectively. The CLSI, EUCAST, and FDA provide guidelines for antimicrobial susceptibility testing, including standardized methods, quality control procedures, and interpretive criteria for assessing the susceptibility of microorganisms to antimicrobial agents.

To study and determine the specific contributions of both *bla*_NDM-1_ and *bla*_CMY-6_ genes to the overall resistance profile, susceptibility to ceftazidime was assessed using commercial E-strips (Etest, Biomerieux, Nürtingen, Germany) according to the manufacturer’s guidelines. This evaluation was conducted using Mueller–Hinton broth alone and with the addition of metallo-β-lactamases (MBL) inhibitor EDTA at a final concentration of 0.4 mM, and cephalosporinases inhibitor 3-amino-phenyl-boronic acid, at a final concentration of 300 µg/mL.

## 4. Conclusions

The concerning resistance patterns observed in this strain, coupled with novel resistance mechanisms in *A. baumannii*, emphasize the necessity for global surveillance to effectively target antimicrobial therapy. Furthermore, this strain represents an emerging threat due to the known potential of ST79 strains to spread across various environments. In summary, *A. baumannii*’s unique ability to evolve and acquire resistance from diverse species highlights the urgent need for ongoing research and clinical efforts to address this public health threat.

## Figures and Tables

**Table 1:** General features of AMA205.

	AMA205 Chromosome	pAMA205_1 Plasmid	pAMA205_2 Plasmid
Size (bp)	4,139,231	16,095	5281
G + C content (%)	50.9	35.32	36.37
Protein-coding sequences (CDSs)	4007	19	7
Insertion sequences	71	0	0
rRNA operons	18	0	0
tRNA	73	0	0

**Table 2:** *Acinetobacter baumannii* AMA205 susceptibility testing.

Antimicrobial Agent	MIC (mg/L)	CLSI ^1^/EUCAST ^2^/FDA ^3^ Breakpoint
CAZ	>256	S ≤ 8; I = 16; R ≥ 32 ^1^
FDC	4 * (IHC)	S ≤ 2; R ≥ 4 ^2^
IMP	96	S ≤ 2; I = 4; R ≥ 8 ^1^
MEM	128	S ≤ 2; I = 4; R ≥ 8 ^1^
AMS	>256	S ≤ 8/4; I = 16/8; R ≥ 32/16 ^1^
AK	>256	S ≤ 16; I = 32; R ≥ 64 ^1^
CN	>1024	S ≤ 4; I = 8; R ≥ 16 ^1^
TIG	0.50	S ≤ 2; R ≥ 8 ^3^

* CAZ: ceftazidime; FDC: cefiderocol; IMP: imipenem; MEM: meropenem; AMS: ampicillin–sulbactam; AK: amikacin; CN: gentamicin; TIG: tigecycline. ^1^ CLSI M100-S34 guidelines [14], ^2^ EUCAST breakpoint tables v.14.0 (https://www.eucast.org/clinical_breakpoints, last accessed 23 October 2024), ^3^ FDA breakpoints (https://www.fda.gov/drugs/development-resources/fda-recognized-antimicrobial-susceptibility-test-interpretive-criteria, last accessed 22 October 2024).

## Data Availability

The Whole Genome Shotgun project was deposited in GenBank with accession numbers CP169298 (chromosome), CP169299, and CP169300 (plasmids).

## Supplementary Materials

The following Supporting Information can be downloaded at: https://www.mdpi.com/article/10.3390/ijms252111424/s1.
